# Agarose Gel Electrophoresis-Based RAPD-PCR—An Optimization of the Conditions to Rapidly Detect Similarity of the Alert Pathogens for the Purpose of Epidemiological Studies

**DOI:** 10.3390/gels8120760

**Published:** 2022-11-22

**Authors:** Tomasz Bogiel, Agnieszka Mikucka, Piotr Kanarek

**Affiliations:** 1 Microbiology Department, Ludwik Rydygier Collegium Medicum in Bydgoszcz, Nicolaus Copernicus University in Torun, 85-094 Bydgoszcz, Poland; 2 Clinical Microbiology Department, Dr Antoni Jurasz University Hospital No. 1 in Bydgoszcz, 85-094 Bydgoszcz, Poland; 3 Department of Microbiology and Food Technology, Faculty of Agriculture and Biotechnology, Bydgoszcz University of Science and Technology, 85-029 Bydgoszcz, Poland

**Keywords:** agarose, agarose gel, alert pathogens, *Enterococcus faecium*, gel electrophoresis, GREfm, RAPD-PCR

## Abstract

Agarose gel electrophoresis is a well-known tool to detect DNA fragments amplified in polymerase chain reaction (PCR). Its usefulness has also been confirmed for epidemiological studies based on restriction fragments length polymorphism (RFLP), usually performed using pulsed-field gel electrophoresis (PFGE). Little is known on the effectiveness for alert-pathogen epidemiological studies of another less time-consuming and costly technique called randomly amplified polymorphic DNA-PCR (RAPD-PCR). Meanwhile, its usefulness is believed to be comparable to RFLP-PFGE. Therefore, the aim of the study was to establish and optimize the conditions of agarose gel electrophoresis following RAPD-PCR for 19 *Enterococcus faecium* strains derived from epidemic outbreaks at intensive care units. An application of different PCR primers, primer combinations, and, in particular, agarose gel concentrations and electrophoresis conditions revealed the usefulness of this relatively fast and inexpensive method based on RAPD-PCR for epidemiological studies without a compulsion to use the specialized equipment necessary for RFLP-PFGE.

## 1. Introduction

The implementation of molecular biology methods and techniques in the rapid detection of *Enterococcus* spp. along with species identification of *E. faecium* can aid in early clinical diagnosis, as well as serve as a predictive tool in hospitals, food processing plants, as well as animal-production-oriented farms. Among the many and varied detection methods in the identification and relatedness investigation of *Enterococcus* spp. (e.g., repetitive-element-sequence-based PCR, plasmid profiling, amplified fragment length polymorphisms), the use of randomly amplified polymorphic DNA RAPD-PCR is considered one of the simplest methods and an increasingly applicable one, especially during clinical outbreaks. In addition, it may be successfully used to screen the presence and similarity of *Enterococcus* spp. isolated from food samples [1,2,3].

*Enterococcus* spp. is a genus of Gram-positive, facultatively anaerobic cocci, adapted during multimillion-year evolution to a commensal existence in the digestive system and, to a minor extent, in the vagina and oral cavity of humans and animals [4,5]. *Enterococcus* spp. are also a leading cause of opportunistic nosocomial infections leading to bacteriemia, endocarditis, urinary tract infections, infections in the abdominal cavity, central nervous system, skin and soft tissue, etc. Among the many species of *Enterococcus* spp., *E. faecium* and *E. faecalis* are the main infectious agents for humans [6,7]. For more than two decades, a change in the *Enterococcus faecalis–Enterococcus faecium* infection ratio has been observed, where *E. faecium* has become the leading agent. One of the explanations for this transition is the high genetic plasticity and the species’ ability to present broad antimicrobial resistance (AMR) [6,8,9].

*E. faecium* (particularly clinical strains) is a bacterium classified as a pathogen of the ESKAPE group (*E. faecium*, *Staphylococcus aureus*, *Klebsiella pneumoniae*, *Acinetobacter baumannii*, *Pseudomonas aeruginosa*, and *Enterobacter* spp.), which contains highly virulent and antibiotic-resistant bacterial alert pathogens [10]. Glycopeptide-resistant *E. faecium* (GREfm) strains, including vancomycin-resistant (VREfm) isolates, are a significant threat to human health, especially as VREfm strains can develop simultaneous resistance to a number of other antimicrobials and last resort drugs such as linezolid and daptomycin, among others, and may easily spread within health-care facilities [11,12,13,14]. 

Therefore, the purpose of this study was to determine the effectiveness of RAPD-PCR—a relatively cheap and fast method for the detection of similarities in one of the important bacterial alert pathogens—for GREfm strains derived from an epidemic outbreak and to optimize agarose gel electrophoresis conditions to increase this technique’s usefulness for rapid epidemiological studies.

## 2. Results and Discussion

### 2.1. RAPD-PCR—Evaluation of an Overall Discriminatory Power

The initial studies were performed with the application of AB106 (Supplementary material, Figure S1A), AB111 (Supplementary material, Figure S1B) and AP4 (Supplementary material, Figure S1C) primers and arbitrarily chosen DNA samples, treated as “a blind control”. The cited parameters [15] were used to evaluate the discriminatory power of the mentioned primers, the RAPD-PCR technique itself and agarose electrophoresis. The achieved results revealed that the number or bands or their pattern obtained in the amplification reaction was not sufficient to distinguish between strains with a great similarity. 

Due to the fact that the obtained patterns may have resulted from the differences in the initial DNA concentration, in the further steps of the study, the amount of DNA added to each reaction was unified to 14 ng/reaction to provide reproducible results.

### 2.2. Discriminatory Power of the Selected Primers

All the DNA samples extracted from the strains and amplified with AP4 primer provided identical patterns (Supplementary material, Figure S1C). Therefore, in the further steps of the study, AP4 primers were combined for RAPD-PCR with all the remaining primers (Supplementary material, Figure S2A–F). 

The initial agarose electrophoresis conditions’ (1.5% agarose, a 1× concentrated Tris-Boric Acid-EDTA, 9 V/cm, 90 min) results presented a sufficient discriminatory power to evaluate strains’ similarity with the application of primers 208 (Supplementary material, Figure S3A), while for 272 primer the possibility to distinguish the patterns of the amplified strains’ DNA increased when the agarose concentration was elevated to 2.5% and the separation time ultimately increased to 150 min (Supplementary material, Figure S3B,C).

**Figure 1:**
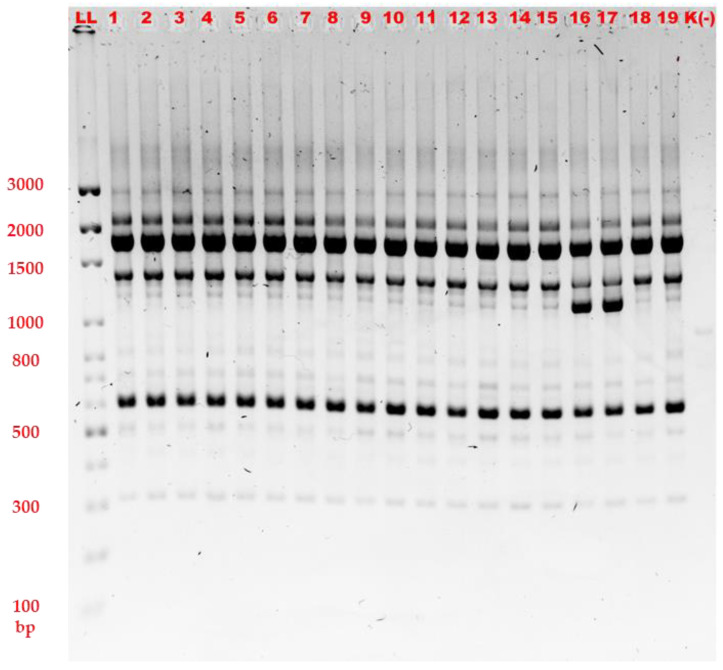
Agarose gel electrophoresis of RAPD-PCR amplification products with AP5 primer, 9 V/cm, 90 min, 1.5% agarose (1–19—numbers of the strains, K(−)—negative control, LL—DNA size marker 100—3000 bp).

For the AP3 primer, the number of bands obtained under standard conditions was too low to provide a satisfactory discriminatory power (Supplementary material, Figure S4); meanwhile, for the AP5 primer, the standard conditions provided a sufficient level of strain discrimination (Figure 1).

### 2.3. Agarose Gel Electrophoresis Optimization

The application of AB106 and AB111 primers presented patterns that gave a satisfactory insight into the molecular similarity of the strains when 12 V/cm and 2.0% agarose were applied (Supplementary material, Figure S5A,B). 

Meanwhile, the more obvious patterns of the strains’ molecular similarity were presented when ARB11 and ERIC1 primers were applied and the agarose concentration was decreased to 1.2% and 1.0%, respectively (Supplementary material, Figure S6A,B).

### 2.4. The Overall Similarity of the Strains

Regardless of the applied primers, an optimization of the agarose concentration and separation conditions revealed that two strains (numbers 16 and 17) were identical and different from the rest of the isolates. Both mentioned strains were derived from the clinical samples collected from the same patient (skin swab and rectal swab, Supplementary material, Table S1). The remaining strains presented the same RAPD-PCR patterns, underlining their origin from an epidemic outbreak and the usefulness of the applied methodology for the study’s purpose.

An application of AP4 combinations with all the other primers in one RAPD-PCR resulted in an increase of discriminatory power for these particular primer pairs, providing results consistent with all the remaining approaches (Supplementary material, Figure S2A–F).

### 2.5. Discussion

The uncontrolled antibiotic application as well as the widespread use of antimicrobials in intensified animal production contribute to the increase in reports of the spread of highly resistant strains of *E. faecium*. They might also be isolated as part of hospital-associated infections (HAIs), including epidemic outbreaks, but also from animal products (such as meat and milk products), which may be the primary source of the horizontal transfer of resistance genes for clinical strains [5,16,17]. In previous studies, the application of the amplification fragment length polymorphism (AFLP) method allowed one to distinguish between two subpopulations of *E. faecium* isolates; the first associated with non-invasive colonization of the gastrointestinal tract and the second linked with nosocomial infections [6]. 

In previous studies conducted by Akpaka et al., the RAPD’s usefulness for typing vancomycin-resistant *Enterococcus* spp. isolates was proven [18]. However, the RAPD-PCR results are sometimes inconclusive, non-repeatable and hard to read due to a lack of interpretation criteria. Nevertheless, the RAPD-PCR is also effective in grouping VRE strains geographically, as well as their environmental distribution (e.g., for veterinary strains) [19]. Moreover, the usefulness of RAPD-PCR has also been proven for vancomycin-resistant *E. faecium* strains by showing that these strains belong to several different patterns and by confirming their polyclonal spread or rather intra-hospital dissemination within healthcare units [20,21,22,23,24].

Wang et al. point out the simultaneous effect of diverse factors affecting the RAPD-PCR reaction. Their study on optimizing the detection of *Enterococcus* spp. from the gut of *Bombyx mori* silkworms demonstrated that the use of Mg^2+^ ions, at a concentration of 3.5 mM, 15 mM of primer, 200 mM dNTPs, 50 ng DNA template, 3U Taq polymerase in 25 µL of reaction mixture with 45 PCR cycles, resulted in the highest amplification effect [25]. However, an optimization of the amplification reaction was not the purpose of the present study, and it is worth highlighting that there is a significant variation between clinical VREfm isolates and environmental strains [26].

The migration rate of DNA molecules in a gel depends on a series of factors: the size of the DNA molecule, type and concentration of agarose, DNA conformation, applied voltage, presence of ethidium bromide or corresponding dyes, electrophoresis buffers, etc. [27]. Meanwhile, the appropriate planning and sufficient optimization of the electrophoresis process can significantly speed up the examination and identification of microorganisms, including the investigation of their molecular similarity, for epidemiological study purposes. For example, the study performed by Saeedi et al. on PFGE methodology demonstrated that defining novel electrophoresis parameters increased the separation rate of smaller and larger DNA fragments and resulted in a reduction in the duration of the detection process for *Enterococcus* spp. [28]. 

Nevertheless, PFGE technique is always time-, cost- and work-consuming when compared to RAPD-PCR applications. Meanwhile, Turabelidze et al. point out that the selection of optimal electrophoresis conditions for VRE significantly reduces the usage of reagents, decreases personnel hand-on time, and results in economic benefits in addition to scientific benefits. This, as a result, may lead to widespread interest in old as well as new detection techniques [7,29].

The concentration of agarose in gel is an important factor that affects the electrophoresis process. Different studies use various concentrations of this polymer in the gel. However, there is a lack of such information in relation to the optimization of RAPD-PCR products’ separation in agarose gel electrophoresis used in epidemiological investigations. In the relevant literature, very few examples of agarose concentration modifications/optimization in molecular studies on *E. faecium* strains can be found. For example, in a study on the identification of *vanA* and *vanB* genes in vancomycin-resistant *Enterococcus* spp. conducted by Ateba et al., 2% agarose gel was used to visualize multiplex PCR results, and electrophoresis was conducted at 60 V for 5 h [30]. On the other hand, a 1.5% agarose gel was selected to examine genes of vancomycin resistance in hospital-based *Enterococcus* spp. strains in a study by Phukan et al. [31]. Another example is the use of 1% agarose gel in an investigation of the spread of VRE in meat and environmental samples in research conducted by Messi et al. [32]. Meanwhile Heuer et al.’s study applied agarose gel at a concentration of 0.8% in 20-h PFGE-type electrophoresis for VRE detection on poultry farms [33]. 

To the best of our knowledge, a great variety of agarose concentrations has been previously applied for molecular studies based on *E. faecium* isolates. However, this aspect has never been studied before for RAPD-PCR with the aim of increasing the usefulness of agarose gel electrophoresis for epidemiological investigation purposes. Meanwhile, considering the simplicity of the methodology, device availability, costs, time, and potential of its use as a routine procedure in epidemiological investigation, such studies seem to be justified, beneficial and even indispensable. The results of epidemiological studies based on agarose gel electrophoresis following RAPD-PCR are repeatable and relatively easy to interpret if the appropriate parameters are applied. A limitation of the study is the relatively small number of strains included; however, for the purpose of the study, this seems to be irrelevant. 

## 3. Conclusions

The results of epidemiological studies based on agarose gel electrophoresis following RAPD-PCR are reliable and relatively easy to interpret if the appropriate parameters are applied. The RAPD-PCR technique is a very useful tool for typing glycopeptide-resistant *E. faecium* isolates and provides reproducible results after optimizations of the agarose-gel electrophoresis conditions.

## 4. Materials and Methods

### 4.1. Bacterial Strains—Origin, Culture and Characteristics

In total, 19 clinical isolates of *E. faecium* strains were included in the study. An initial strain identification was based on typical growth on a selective medium (DCO, bioMérieux, Lyon, France). The final identification was obtained using the MALDI-TOF MS method via the MALDI Biotyper apparatus (Bruker, Mannheim, Germany). All of the tested strains were isolated and banked at the Clinical Microbiology Laboratory of Dr. Antoni Jurasz University Hospital No. 1 in Bydgoszcz. The study material came from patients of the Anesthesiology and Intensive Care Unit and Clinical Unit of Anaesthesiology and Intensive Care with Cardiac Anaesthesiology Division. The detailed origin of the strains and their susceptibility profiles are shown in the Supplementary material (Table S1).

### 4.2. Bacterial DNA Isolation

The DNA extraction was carried out using a Genomic Mini kit (A&A Biotechnology, Gdynia, Poland) in accordance with the manufacturer’s instructions, with the addition of mutanolysin (Sigma) and lysozyme (Sigma) to improve DNA isolation efficiency strains. Prior to their use for the study, all the DNA samples were kept in a 4 °C freezer.

### 4.3. RAPD-PCR

The similarity of the strains was performed by RAPD-PCR in a separate reaction for each primer. The amplification procedure was carried out as previously described, and the primers were chosen based on the published references [15]. The reactions were carried out in 0.2 mL test tubes (Eppendorf, Germany) with a final volume of 20 μL. Briefly, Taq polymerase was used with a total activity of 1 U per reaction in a 1× concentrated polymerase buffer with MgCl_2_ at a final concentration of 2 mM (Go Taq G2 Polymerase, Promega, Germany) and deoxynucleotide triphosphates (dNTPs) set at a final concentration of 200 μM (Promega, Germany). Primers were used at a final concentration of 1.5 μM (Integrated DNA Technologies, Coralville, IA, USA and Genomed, Poland). The primers’ sequences and their melting temperature are presented in Table 1. In the amplification procedure, a thermal cycler GeneAmp^®^ PCR System 2700 (Applied Bio-systems, Foster City, CA, USA) was applied, and the mixture was subjected to 94 °C for 5 min of initial denaturation, 44 cycles of 94 °C for 30 s, 35 °C for 1 min, and 72 °C for 2 min, followed by 10 min at 72 °C.

### 4.4. Agarose Gel Electrophoresis—Standard Conditions and Their Optimization

Initially, all the RAPD-PCR products were separated by electrophoresis in 1.5% agarose (Sigma, St. Louis, MO, USA) stained with Midori Green Advance DNA Stain solution (ABO, Gdańsk, Poland) in a 1× concentrated Tris-Boric Acid-EDTA (TBE, Bio-Rad, Feldkirchen, Germany) running buffer in MINI SUB DNA CELL and SUB-CELL GT (Bio-Rad, Feldkirchen, Germany) devices at 9 V/cm within 90 min. Their images were captured and processed in the Gel Doc 2000 system using the Quantity One (Bio-Rad, Feldkirchen, Germany) program. Fragments of DNA were separated along with the size marker (100–3000 bp DNA size marker, Solis BioDyne, Estonia) in every separation round.

Initially the RAPD-PCR was performed with an application of 10-bp primers: AB106, AB111, as well as AP4 primer, recommended by Barbier et al. [15], on arbitrarily chosen DNA isolates extracted from random *E. faecium* isolates (all three presented in Supplementary material, Figure S1). The first two primers presented a sufficient discriminatory power and served for further investigation on all of the strains included in the study. Since the latter primer did not provide a sufficient discriminatory power, it has been combined with all the remaining primers in the same reaction.

Secondly, other primers (namely 208 and 272) were involved for the study’s purpose. Both were the same length (10 bp), and the same agarose separation parameters were applied (Supplementary material, Figure S3).

Thirdly, other 10-bp primers (namely AP3 and AP5) were involved for the study’s purpose, and the same agarose separation parameters were applied (Supplementary material, Figure S4 and Figure 1).

Further studies involved an application of AB106 and AB111 primers (both 10-bp) with their amplification products’ separation at an increase: voltage (12 V/cm), agarose concentration (2.0%) and time—up to 105 min (Supplementary material, Figure S5).

Finally, ERIC1 (22-bp) and ARB11 (10-bp) primers were applied in the study with RAPD-PCR product separation in agarose concentrations of 1.2% and 1.0%, respectively (Supplementary material, Figure S6).

In the end, combinations of AP4 and other primers for amplification rounds were applied (Supplementary material, Figure S2).

Further procedures involved changes in agarose concentration (1.0–2.7% agarose), voltage (up to 12 V/cm) and time (up to 150 min), while also introducing subsections under separation conditions (12 V/cm for 45 min and 9 V/cm for the subsequent 45 min).

### 4.5. Quality Assurance and Quality Control

Selective solid medium for bacteria culture—an ability to grow was validated using the following strains: *Escherichia coli* ATCC 25922, *E. faecalis* ATCC 29212, *E. faecium* ATCC 19434, and *Streptococcus pyogenes* ATCC 19615.

Strain identification via mass spectrometry—an analysis was validated with an internal standard reference of *E. coli*; the species identification score was accepted only for those strains that showed a score value between 2.00 and 3.00 (high-confidence identification); other strains were rejected.

DNA isolation—in order to improve the isolation efficacy of bacterial DNA, a commercially available kit was used, and the procedure was performed in accordance with the manufacturer’s instructions; recombinant mutanolysin and lysozyme enzymes (which exhibit synergistic activity) were added.

Incubation time and temperature—these were strictly controlled at each step of the research.

Validation of the discriminatory power of the primers—since one of the RAPD-PCR primers did not display a sufficient discriminatory role in the randomly selected *Enterococcus* spp. strains tested, it was excluded from further study steps.

The use of a wide range of electrophoresis conditions in the RAPD-PCR-based methodology, along with quality procedures, allows for the selection of the most optimal solution.

## Figures and Tables

**Table 1:** Specification of the primers applied in this study.

Primer Name	Manufacturer	Primer Sequence 5′→3′	Tm
AP3	Genomed	TCACGATGCA	30.0 °C
AP4		TCACGCTGCA	32.0 °C
AP5		TCACGCTGCG	34.0 °C
AB106		TGCTCTGCCC	34.0 °C
AB111		GTAGACCCGT	32.0 °C
ARB11		CTAGGACCGC	34.0 °C
ERIC1		ATGTAAGCTCCTGGGGATTCAC	54.8 °C
208	Integrated DNA Technologies	ACGGCCGACC	45.5 °C
272		AGCGGGCCAA	43.7 °C

## Data Availability

The data presented in this study are available on request from the corresponding author.

## Supplementary Materials

The following supporting information can be downloaded at: https://www.mdpi.com/article/10.3390/gels8120760/s1, Table S1: The detailed origin of *E. faecium* strains included in the study and their susceptibility profiles; Figures S1–S6: Agarose-gel electrophoresis of RAPD-PCR amplification products separated under the following parameters.
