# Supplementary material for: Agarose Gel Electrophoresis-Based RAPD-PCR—An Optimization of the Conditions to Rapidly Detect Similarity of the Alert Pathogens for the Purpose of Epidemiological Studies
Source: Gels. 2022 Nov 22;8(12):760. doi: 10.3390/gels8120760 (PMC9778174; doi:10.3390/gels8120760)

gels MDPI

Supplementary material

Agarose gel electrophoresis-based RAPD-PCR—An optimization of the conditions to rapidly detect similarity of the alert pathogens for the purpose of epidemiological studies

Tomasz Bogiel [1,2], Agnieszka Mikucka [1,2] and Piotr Kanarek [3]

[1] Microbiology Department, Ludwik Rydygier Collegium Medicum in Bydgoszcz, Nicolaus Copernicus University in Torun, Poland; a.mikucka@cm.umk.pl (A.M.)
[2] Clinical Microbiology Department dr Antoni Jurasz University Hospital No 1 in Bydgoszcz, Poland;
[3] Department of Microbiology and Food Technology, Faculty of Agriculture and Biotechnology, Bydgoszcz University of Science and Technology, Bydgoszcz, Poland; piokan004@pbs.edu.pl (P.K.)
* Correspondence: t.bogiel@cm.umk.pl; Tel.: +48 52 585 44 80

Table S1. The detailed origin of *E. faecium* strains included into the study (n = 19) and their susceptibility profiles

Strain No.	Patient No.	Specimen type	Department /unit	Ampicillin	Amoxicillin/ clavulanate	Piperacillin	Piperacillin/ tazobactam	Imipenem	Gentamicin	Streptomycin	Tigecycline	Vancomycin	Teicoplanin	Linezolid
1	1	Skin swab	Department of Anaesthesiology and Intensive Care	R	R	R	R	R	S	R	S	R	R	S
2	1	Wound swab	Department of Anaesthesiology and Intensive Care	R	R	R	R	R	S	R	S	R	R	S
3	2	Bronchoalveolar lavage	Department of Anaesthesiology and Intensive Care	R	R	R	R	R	R	R	S	R	R	S
4	3	Wound swab	Department of Anaesthesiology and Intensive Care	R	R	R	R	R	R	R	S	R	R	S
5	4	Peritonel fluid	Clinical Unit of Anaesthesiology and Intensive Care with Cardiac Anaesthesiology Division	R	R	R	R	R	R	R	S	R	R	S

6	4	Rectal swab	Clinical Unit of Anaesthesiology and Intensive Care with Cardiac Anaesthesiology Division	R	R	R	R	R	R	R	S	R	R	S
7	4	Skin swab	Clinical Unit of Anaesthesiology and Intensive Care with Cardiac Anaesthesiology Division	R	R	R	R	R	R	R	S	R	R	S
8	5	Rectal swab	Clinical Unit of Anaesthesiology and Intensive Care with Cardiac Anaesthesiology Division	R	R	R	R	R	R	R	S	R	R	S
9	5	Skin swab	Clinical Unit of Anaesthesiology and Intensive Care with Cardiac Anaesthesiology Division	R	R	R	R	R	R	R	S	R	R	S
10	2	Wound swab	Department of Anaesthesiology and Intensive Care	R	R	R	R	R	R	R	S	R	R	S
11	2	Throat swab	Department of Anaesthesiology and Intensive Care	R	R	R	R	R	R	R	S	R	R	S
12	2	Skin swab	Department of Anaesthesiology and Intensive Care	R	R	R	R	R	R	R	S	R	R	S
13	2	Rectal swab	Department of Anaesthesiology and Intensive Care	R	R	R	R	R	R	R	S	R	R	S
14	6	Rectal swab	Clinical Unit of Anaesthesiology and Intensive Care with Cardiac Anaesthesiology Division	R	R	R	R	R	R	R	S	R	R	S
15	6	Skin swab	Clinical Unit of Anaesthesiology and Intensive Care with Cardiac Anaesthesiology Division	R	R	R	R	R	R	R	S	R	R	S
16	7	Rectal swab	Department of Anaesthesiology and Intensive Care	R	R	R	R	R	R	R	S	R	R	S
17	7	Skin swab	Department of Anaesthesiology and Intensive Care	R	R	R	R	R	R	R	S	R	R	S
18	8	Peritoneal fluid	Department of Anaesthesiology and Intensive Care	R	R	R	R	R	S	R	S	R	R	S
19	9	Skin swab	Department of Anaesthesiology and Intensive Care	R	R	R	R	R	R	R	S	R	R	S
			Resistant strains [%]	100	100	100	100	100	84.2	100	0	100	100	0
			Susceptible strains [%]	0	0	0	0	0	15.8	0	100	0	0	100

R – resistant strains, S – sensitive strains

gels

MDPI

Figure S1. Agarose gel electrophoresis of RAPD-PCR amplification products separated under the following parameters (arbitrarily chosen DNA samples, treated as "a blind control")

A. AB106 primer, 9 V/cm, 90 min, 1.5% agarose (the first attempt)

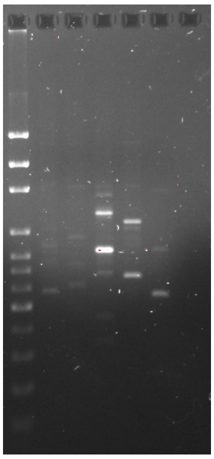

B. AB111 primer, 9 V/cm, 90 min, 1.5% agarose (the second attempt)

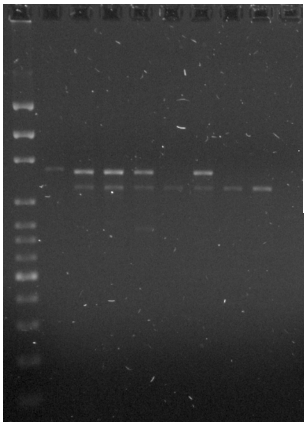

C. AP4 primer, 9 V/cm, 90 min, 1% agarose (the third attempt)

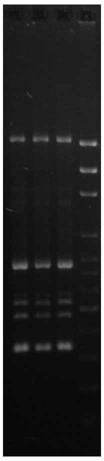

Figure S2. Agarose gel electrophoresis of RAPD-PCR amplification products with AP4 and the second primer under the following parameters (1-19 – numbers of the strains, K(-) – negative control, LL – DNA size marker 100 – 3.000 bp)

A. combination of AP4 and 208 primers, 12 V/cm, 135 min, 2.7% agarose

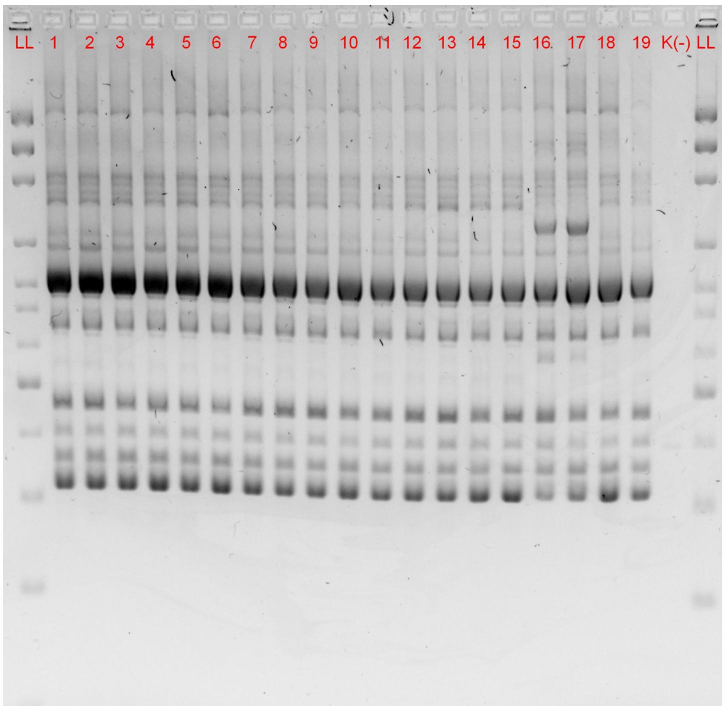

B. combination of AP4 and AP5 primers, 9 V/cm, 135 min, 2.5% agarose

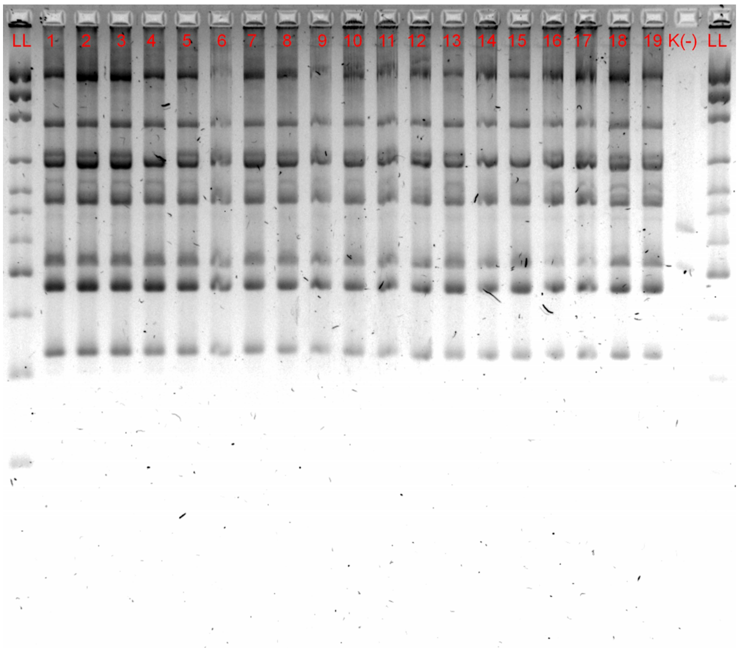

C. combination of AP4 and AB106 primers, 9 V/cm, 90 min, 1.2% agarose

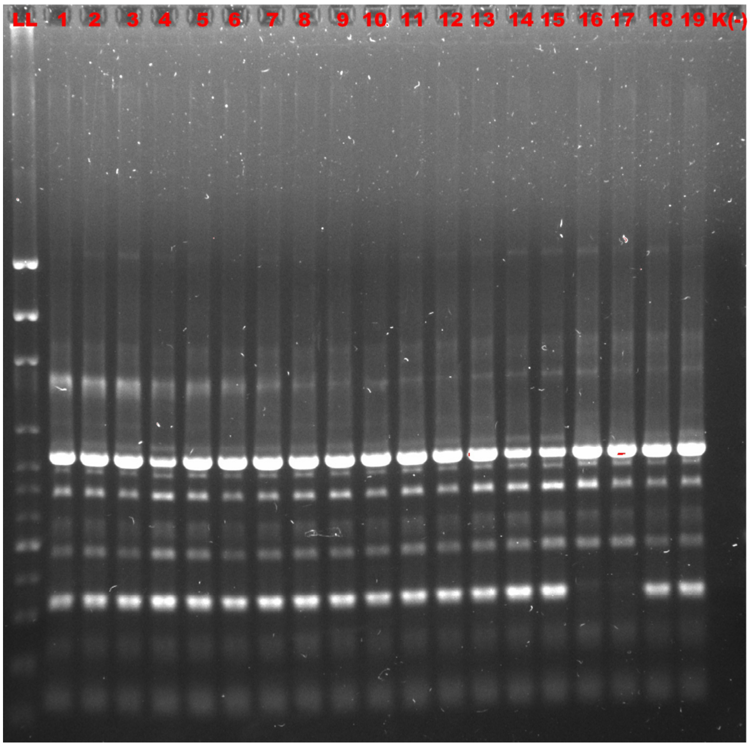

D. combination of AP4 and AB111 primers, 9V/cm, 90 min, 1.2% agarose

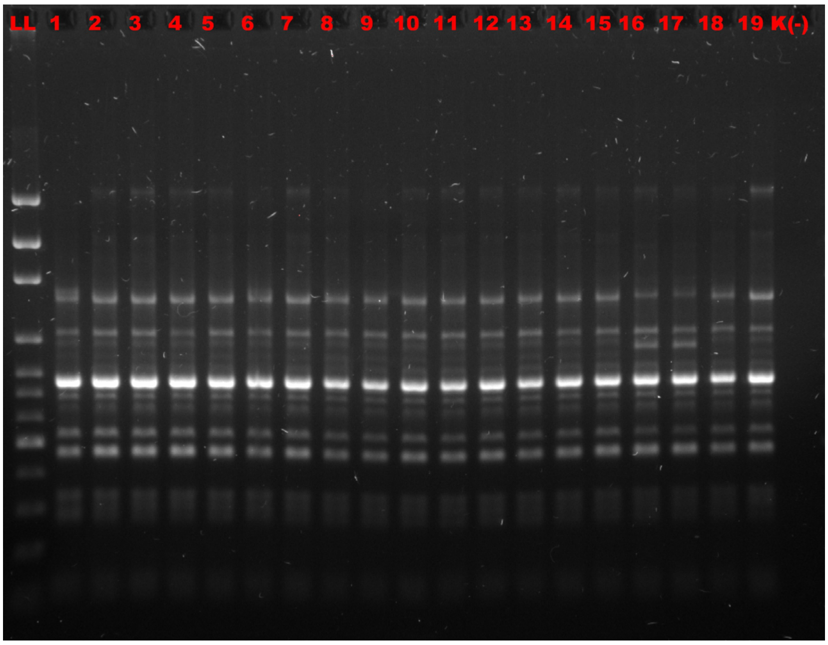

E. combination of AP4 and ERIC1 primers, 120 V – 45 min and 9V/cm - 45 min, 2.0% agarose

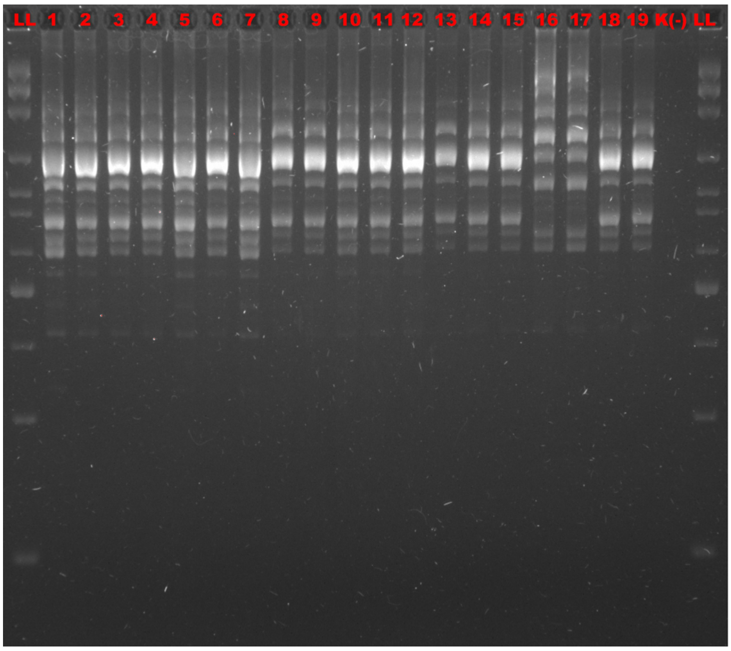

F. combination of AP4 and ERIC1 primers, 9V/cm, 75 min, 1.2% agarose

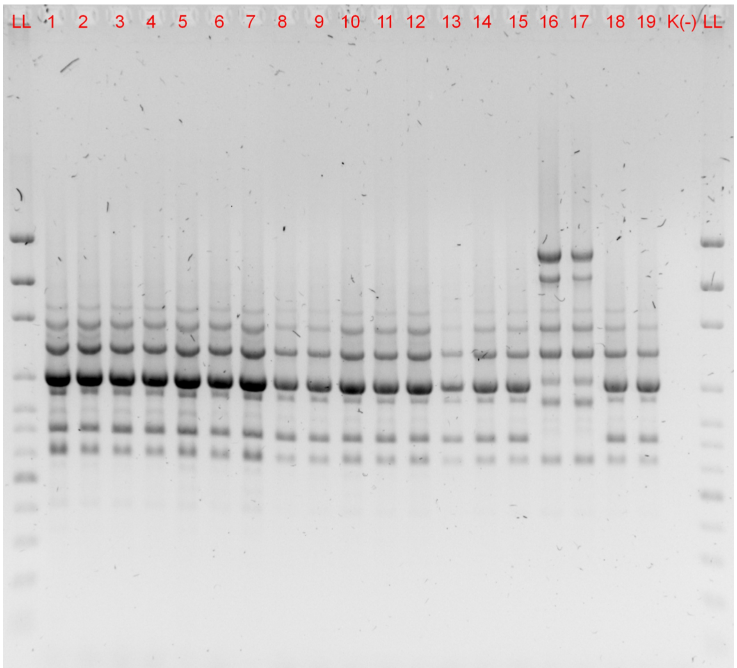

Figure S3. Agarose gel electrophoresis of RAPD-PCR amplification products with 208 and 272 primers under the following parameters (1-19 – numbers of the strains, K(-) – negative control, LL – DNA size marker 100 – 3.000 bp)

A. 208 primer, 9 V/cm, 90 min, 1.5% agarose

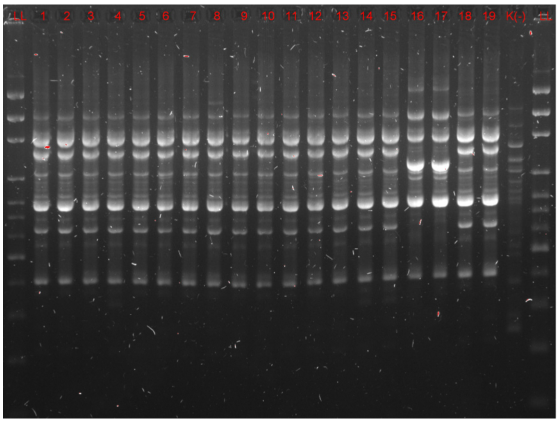

B. 272 primer, 9 V/cm, 135 min, 2.5% agarose

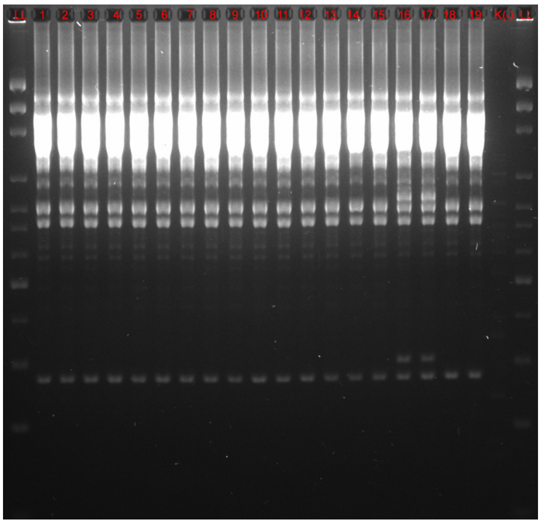

C. 272 primer, 9 V/cm, 150 min, 2.5% agarose

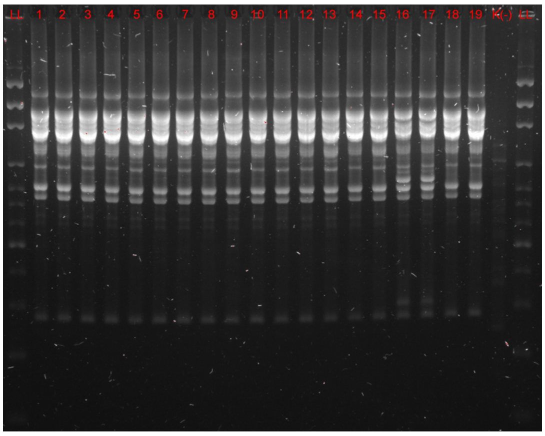

Figure S4. Agarose gel electrophoresis of RAPD-PCR amplification products with AP3 primer under the following parameters (1-19 – numbers of the strains, K(-) – negative control, LL – DNA size marker 100 – 3.000 bp)

AP3 primer, 9 V/cm, 90 min, 1.5% agarose

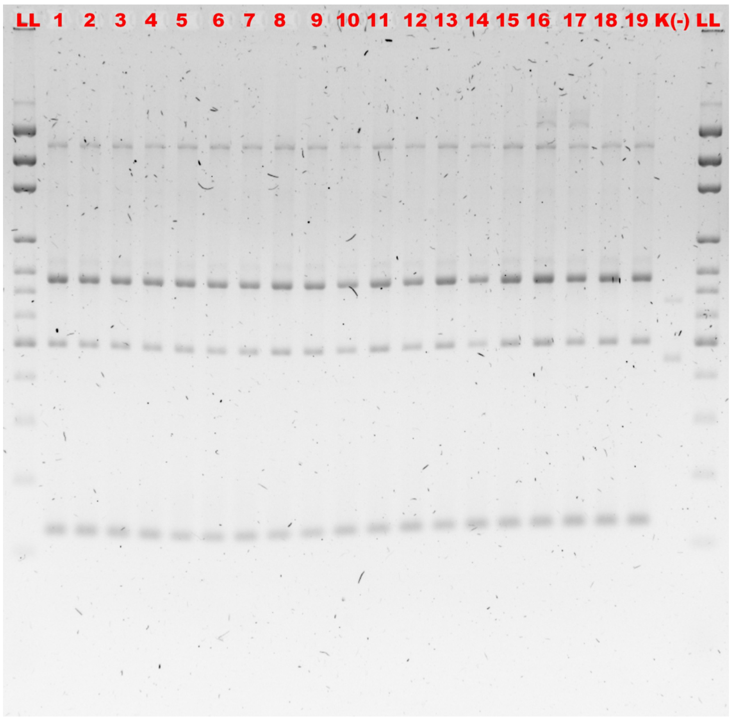

Figure S5. Agarose gel electrophoresis of RAPD-PCR amplification products with AB106 and AB111 primers under the following parameters (1-19 – numbers of the strains, K(-) – negative control, LL – DNA size marker 100 – 3.000 bp)

A. AB106 primer, 12 V/cm, 105 min, 2.0% agarose

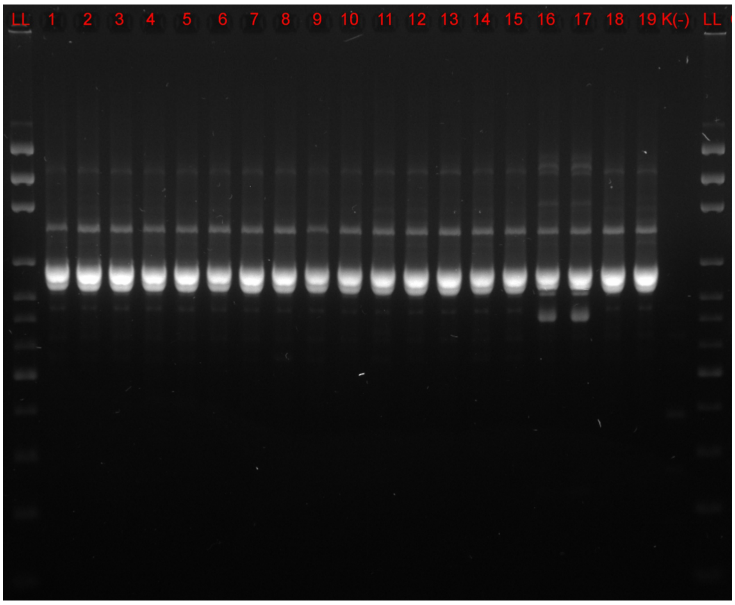

B. AB111 primer, 12 V/cm, 90 min, 2.0% agarose

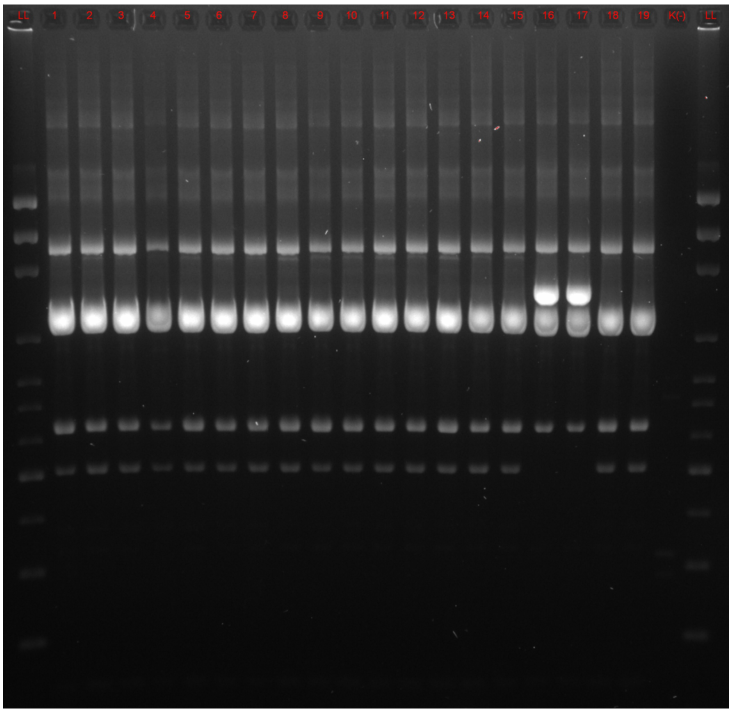

Figure S6. Agarose gel electrophoresis of RAPD-PCR amplification products with ARB11 and ERIC1 primers under the following parameters (1-19 – numbers of the strains, K(-) – negative control, LL – DNA size marker 100 – 3.000 bp)

A. ARB11 primer, 9 V/cm, 90 min, 1.2% agarose

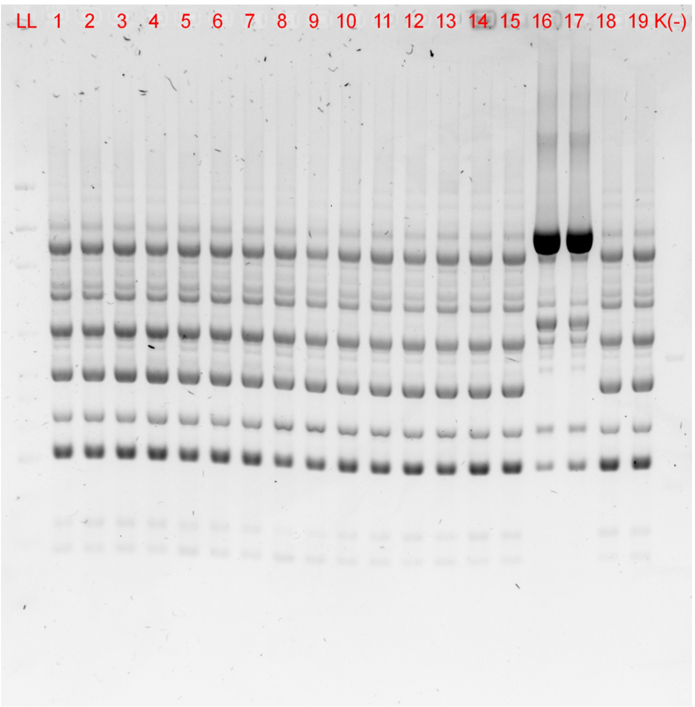

B. ERIC1 primer, 9 V/cm, 90 min, 1.0% agarose

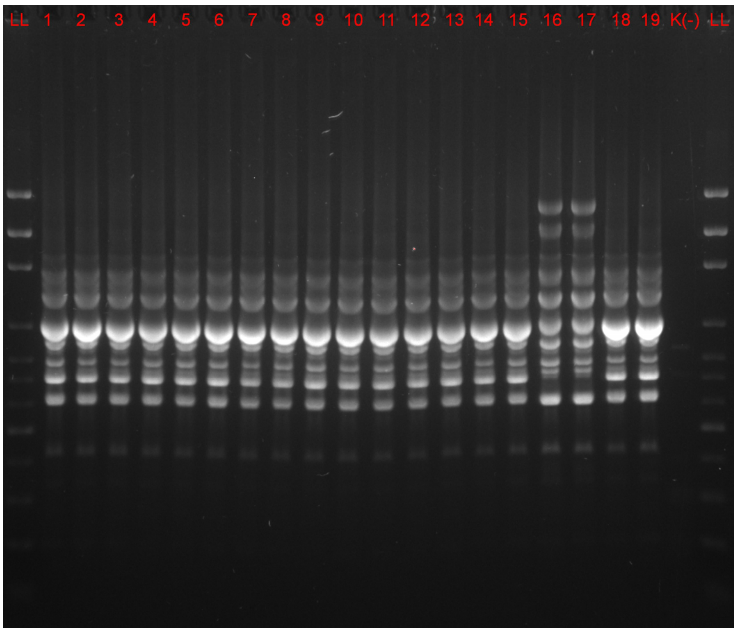

Supplement: Supplementary file 1 [file gels-08-00760-s001.zip › gels-2032881-Supplementary.pdf]
